# Piglet Gut and in-Barn Manure from Farms on a Raised without Antibiotics Program Display Reduced Antimicrobial Resistance but an Increased Prevalence of Pathogens

**DOI:** 10.3390/antibiotics10101152

**Published:** 2021-09-24

**Authors:** Samuel M. Chekabab, John R. Lawrence, Alvin C. Alvarado, Bernardo Z. Predicala, Darren R. Korber

**Affiliations:** 1 Food and Bioproduct Sciences, University of Saskatchewan, 51 Campus Drive, Saskatoon, SK S7N 5A8, Canada; smc349@mail.usask.ca; 2 Prairie Swine Centre Inc., Box 21057, 2105—8th Street East, Saskatoon, SK S7H 5N9, Canada; alvin.alvarado@usask.ca (A.C.A.); bernardo.predicala@usask.ca (B.Z.P.); 3 Environment and Climate Change Canada, 11 Innovation Blvd., Saskatoon, SK S7N 3H5, Canada; john.lawrence2@canada.ca; 4 Chemical and Biological Engineering, University of Saskatchewan, 57 Campus Drive, Saskatoon, SK S7N 5A9, Canada

**Keywords:** RWA, AMR, WGS metagenomics, swine industry, piglet gut, manure

## Abstract

In response to new stringent regulations in Canada regarding the use of antibiotics in animal production, many farms have implemented practices to produce animals that are raised without antibiotics (RWA) from birth to slaughter. This study aims to assess the impact of RWA production practices on reducing the actual total on-farm use of antibiotics, the occurrence of pathogens, and the prevalence of antimicrobial resistance (AMR). A 28-month longitudinal surveillance of farms that adopted the RWA program and conventional farms using antibiotics in accordance with the new regulations (non-RWA) was conducted by collecting fecal samples from 6-week-old pigs and composite manure from the barn over six time points and applying whole-genome sequencing (WGS) to assess the prevalence of AMR genes as well as the abundance of pathogens. Analysis of in-barn drug use records confirmed the decreased consumption of antibiotics in RWA barns compared to non-RWA barns. WGS analyses revealed that RWA barns had reduced the frequency of AMR genes in piglet feces and in-barn manure. However, metagenomic analyses showed that RWA barns had a significant increase in the frequency of pathogenic *Firmicutes* in fecal samples and pathogenic *Proteobacteria* in barn manure samples.

## 1. Introduction

The contamination of terrestrial and aquatic ecosystems and associated food webs with antimicrobial drugs constitutes a public health concern of global proportions. Worldwide, the yearly usage of antibiotics exceeds 1 × 10^5^ tons [1]. Levels of antimicrobial use vary significantly between countries [2,3]. In Canada, a total of 88% of antimicrobially active ingredients sold in 2014 were for use in producing food animals [3]. There appears to be a similar trend observed around the world, where an estimated 73% of all therapeutic antimicrobials are administered to farm animals [4]. Over the past 75 years, the use of antibiotics for food production (e.g., agriculture and aquaculture) has consistently increased, correlating with the discoveries of new antimicrobial drugs over the same time frame [5,6,7]. One outcome of increasing antibiotic use, in general, has been the occurrence of antimicrobial resistance (AMR), where bacteria undergo and consequently exhibit reduced susceptibility to antibiotics. Over the past decade, scientific communities, along with global public health agencies, have raised concerns about AMR and the major challenges faced by health care, which require immediate attention to ensure a continuous and stable supply of clinically relevant drugs for therapeutic use. 

The Canadian hog industry is a major economic driver of the agri-food sector. Overall, the antibiotics that are extensively used by pig producers belong to two main classes: tetracyclines and macrolides [8]. It is notable that most drugs (approximately 75%) have historically been used for prophylactic animal disease control and growth promotion. This means that even though pathogens may be targeted by specific drugs, many non-pathogenic bacteria are also impacted and thus can contribute to the expansion of the AMR pool. In order to mitigate the risks posed by the AMR threat, more stringent rules governing the use of clinical drugs for the treatment of sick animals have been in place in Canada since December 2018 [9]. These new regulations require that the use of medically important antimicrobials in animal production should be under veterinary oversight, ensuring that they are not used prophylactically or as growth promoters in animal feed. Consequently, many Canadian producers have proactively implemented procedures wherein animals are raised without antibiotics (RWA) from birth to slaughter.

In pig production, the nursery stage is key in terms of animal development. After being with the sows from birth, piglets are typically weaned from the sows at about 3–4 weeks of age and then moved to the nursery, where they are kept for about 6–8 weeks before progressing to the grower stage. The stress of weaning and the rapid developmental changes that occur during this period renders the piglets highly susceptible to diseases and health challenges; thus, most antimicrobial drugs are typically administered in the nursery, making it an ideal stage to investigate the impacts of RWA [10]. On the other hand, manure in pig barns is composed of feces and urine from animals of all ages, and it also typically contains livestock bedding, wasted water, and feed. Pig manure is a valuable fertilizer that contains nutrients such as nitrogen, phosphorus, and potassium as well as micronutrients such as copper, manganese, and zinc, among others. Manure from pig barns is also an excellent source of organic matter and can help improve soil quality; hence, barn manure is regularly applied on croplands. As such, it comprises a major link in the spread of AMR and represents a significant reservoir for bacterial pathogens to survive in the ecosystem and potentially sustain the threat of AMR. 

In this paper, we compare the temporal effects of RWA versus non-RWA (conventional) operations on antimicrobial resistance gene (ARG) prevalence and pathogen abundance in the gut of piglets and in-barn manure. Our approach focused on monitoring antibiotic usage, antibiotic resistance, and the prevalence of pathogens. Analysis of in-barn records confirmed that RWA measures were effective in reducing the utilization of antibiotics. Whole-genome sequence (WGS) analysis demonstrated that RWA measures were also significantly correlated with an increase in pathogenic *Firmicutes* in piglet gut as well as pathogenic *Proteobacteria* in manure. In contrast, resistome profiling revealed a positive impact of RWA measures that correlated with reduced ARGs. 

## 2. Results

### 2.1. Decreased Antibiotic Utilization Changed the Occurrence of Scours and Injury in RWA Piglets

**Figure 1:**
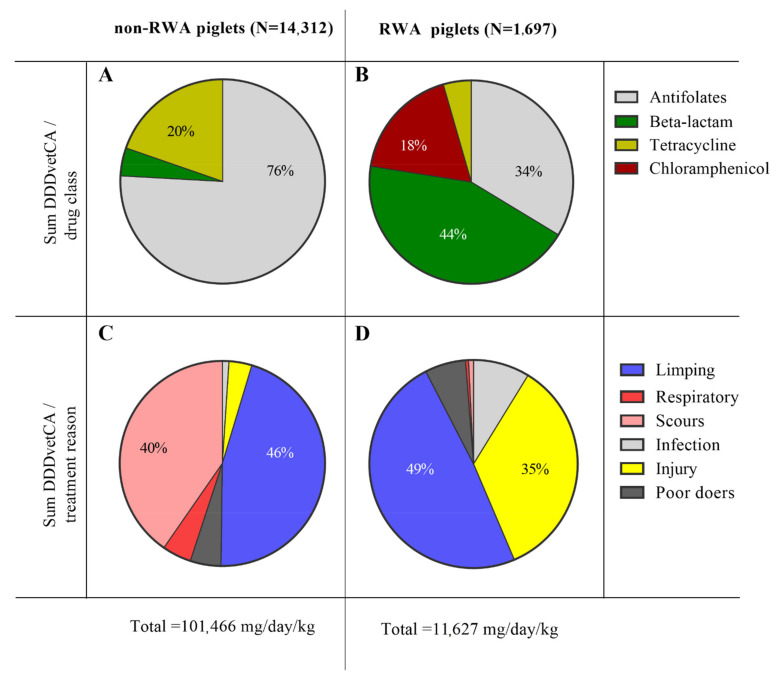
Summary of records from non-RWA and RWA participating farms showing the total DDDvetCA value of antibiotic classes administered to piglets (**A**,**B**), along with clustered diseases and treatment reasons (**C**,**D**).

Documentation of usage of drugs, together with all drug-related metadata, at four participating farms was conducted regularly from August 2018 to November 2020 (28 months). These monthly records included drug dosage, number and age of treated animals, cause of treatment, location in the barn, as well as the date of drug administration. Data on antibiotic type and dosages were normalized using the number of piglets treated during the surveillance period in order to report the values as DDDvetCA, which is the Canadian defined daily dose (average labeled dose) in milligrams per kilogram pig weight per day (mg drug/Kg animal/day), in accordance with the Canadian Integrated Program for Antimicrobial Resistance Surveillance 2016 report [11]. The majority of antibiotics given to piglets during these 28 months belonged to four classes: antifolates (Trimidox), β-lactams (Penicillin G, Ampicillin, Ceftiofur), tetracycline (Biomycin), and chloramphenicol (Nuflor) (Table S1). On average, a total of 5.3 Kg of antibiotics was administered to 14,312 non-RWA piglets, corresponding to 101,466 mg/day/Kg cumulative DDDvetCA (Figure 1C). RWA piglets (N = 1697), however, received a total of 0.86 Kg of antibiotics, corresponding to an 11,627 mg/day/Kg cumulative DDDvetCA value (Figure 1D). Thus, we estimated that total antibiotics usage was reduced by nearly 9-fold in RWA farms compared to non-RWA farms. Once treated with antibiotics, the piglets are taken out of the RWA program and housed in a parallel non-RWA stream. Piglets in non-RWA farms were treated with antifolates (64% of total antibiotics administered), β-lactams (11%), and tetracycline (25%) (Table S1), which respectively corresponded to 76%, 4%, and 20% of the cumulative DDDvetCA value (Figure 1A). In contrast, RWA piglets were treated with antifolates (30% of total antibiotics administered), β-lactams (54%), chloramphenicol (13%), and tetracycline (4%) (Table S1), which respectively corresponded to 34%, 44%, 18%, and 4% of the cumulative DDDvetCA value (Figure 1B). 

Drug documentation records also captured the reason for drug treatments and were accordingly entered into our barn health meta-database, which included 33 separate treatment categories, clustered based on similarities and synonyms of clinical symptoms [12]. Overall, the six most prevalent illnesses and reasons for treatment recorded during the 28-month study period included limping, scours, injury, respiratory impairment, infection, and poor-doers (Figure 1C,D). These clustering procedures allowed the monitoring and comparison of treatment reasons in the RWA barns with non-RWA conventional operations. Limping piglets were the most commonly treated in both non-RWA (46% DDDvetCA) and RWA barns (49% DDDvetCA). Limping symptoms in non-RWA piglets were treated with antifolates or tetracycline, whereas RWA piglets received either antifolates or chloramphenicol. Scours were the second most important symptom observed in the non-RWA group (40% DDDvetCA), and pigs exhibiting scours were treated for up to 3 days with an anti-inflammatory agent combined with either antifolates or tetracycline. RWA piglets had substantially fewer treatments for symptoms of scours; however, 35% of their total DDDvetCA value was administered for injury symptoms, including scratches and visible wounds.

### 2.2. Pathogenic Firmicutes and Proteobacteria were More Prevalent in Barn Manure and Piglet Fecal Samples from RWA Barns

To determine the prevalence of pathogens in the samples, the initial focus of the analysis was the taxonomic display of the frequencies of bacteria at the phylum and class levels, along with species/strain level determinations. Bacterial sequences accounted for nearly 99% of all sequenced reads, matching the k-mer nucleotide markers. Analysis through the CosmosID bioinformatics platform resulted in the identification of 175 bacterial strains belonging to 137 species, 79 genera, 23 classes, and 11 phyla (Figure S1). The piglets’ fecal microbiomes were comprised mainly of *Firmicutes* (71–83%) and *Bacteriodetes* (12–20%). The barn manure collected from the transfer pit, however, showed more diversity, with up to 4 phyla having >5% relative abundance, including *Firmicutes* (56–64%), *Bacteriodetes* (13–20%), *Actinobacteria* (4–10%), and *Proteobacteria* (6–13%).

**Figure 2:**
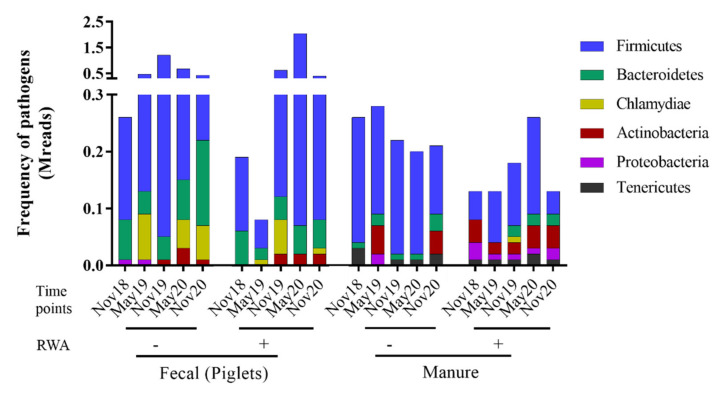
Metagenomic taxonomic profiles at the phylum level from piglet feces and barn manure samples collected from non-RWA − (minus sign) and RWA + (plus sign). The stacked bars represent the averaged frequency of the major bacteriomes from each type of barn.

**Figure 3:**
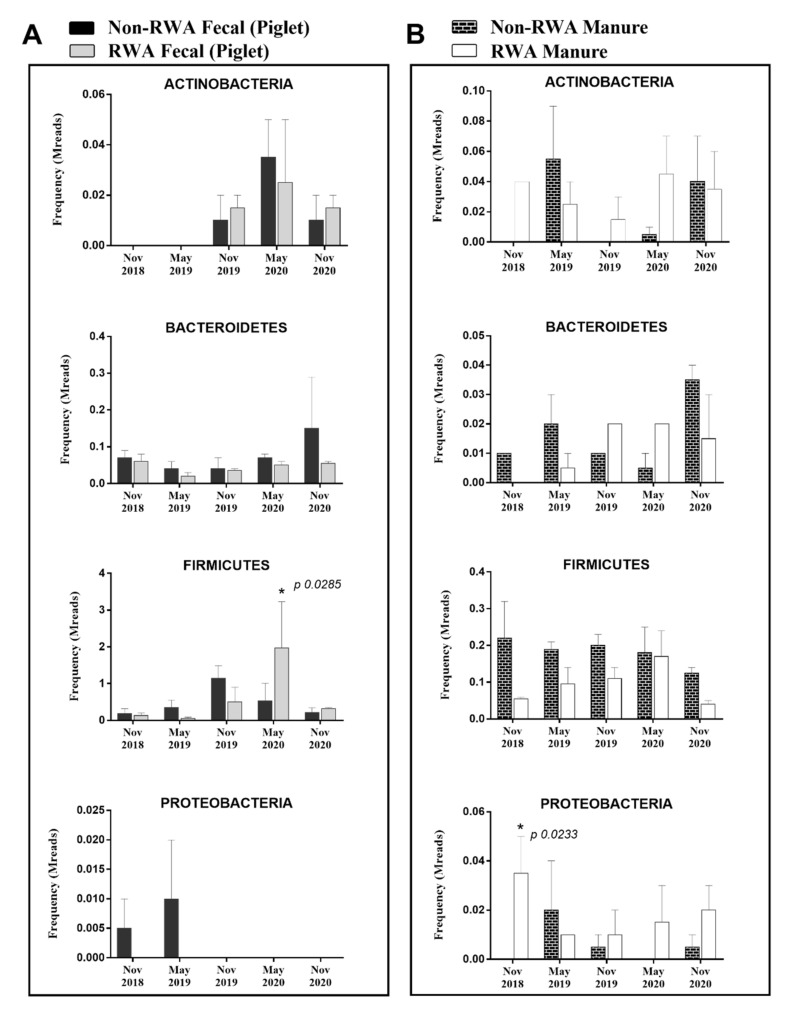
Pathogen prevalence in piglet feces (**A**) and barn manure (**B**) samples. The stacked bars represent the averaged frequency of the pathogens obtained from all bacteriome phyla by extracting subsets of human and/or animal RG2 species.

The pathome, or pathogen prevalence, is represented by the subset of human and/or animal risk group (RG) 2 and RG3 organisms extracted from the data of total species/strains present in the microbiome. From our analysis, 91 and 122 pathogenic strains were found in the piglet feces and barn manure, respectively (see Tables S2 and S3). The total prevalence of pathogens (sequence frequency) was comparable between the RWA and non-RWA barns for piglet feces and barn manure, with an overall reduction in the RWA samples (Figure 2). However, at the phylum level, RWA-piglet feces exhibited a significant increase in the frequency of pathogenic *Firmicutes* (Figure 3A), which included the classes Negativicutes (*Acidaminococcus intestini*, *Mitsuokella multacida*), Bacilli (*Streptococcus equinus*), and Clostridia (*Faecalibacterium prausnitzii*) (Table S2). Additionally, RWA-barn manure showed a significant increase in the frequency of pathogenic *Proteobacteria* (Figure 3B), which included the classes α-proteobacteria (*Paracoccus sanguinis*), γ-proteobacteria (*Escherichia coli* O157:H7 str. 2011EL-2287, *Actinobacillus minor*, *Proteus vulgaris*, *Proteus penneri*, *Leclercia adecarboxylata*, *Proteus mirabilis*, *Serratia liquefaciens*, *Serratia marcescens, Vibrio cholerae*), and β-proteobacteria (*Sutterella wadsworthensis*, *Oligella urethralis*) (Table S3).

### 2.3. RWA Practices Reduced AMR in Piglet Gut and Barn Manure

**Figure 4:**
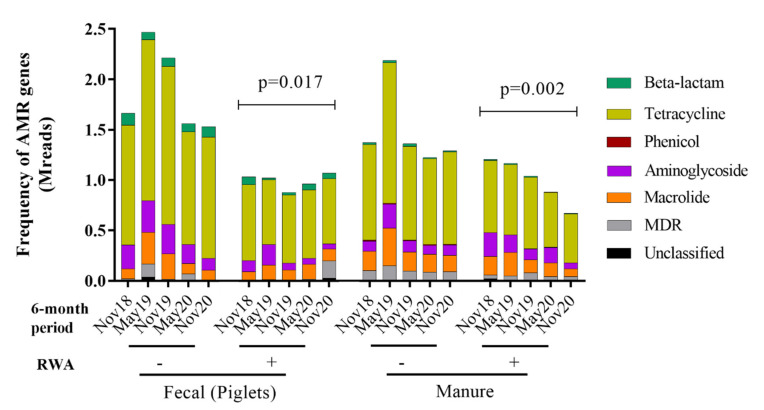
Metagenomic resistome showing the frequency of antibiotic resistance genes (ARGs) clustered into 6 classes—tetracycline, aminoglycosides, macrolides, phenicol, β-lactams, and multi-drug resistance (MDR)—and collected from non-RWA − (minus sign) and RWA + (plus sign) piglet feces and barn manure samples.

**Figure 5:**
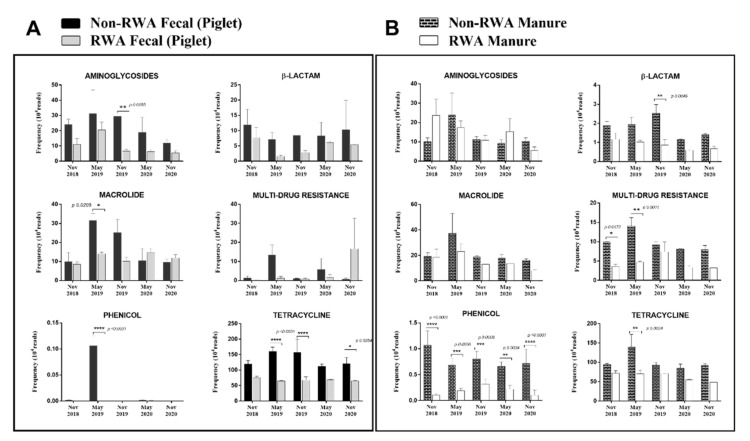
Comparison of non-RWA − (minus sign) and RWA + (plus sign) resistomes from piglet feces (**A**) and barn manure (**B**). The frequency of antibiotic resistance genes (ARGs) clustered into 6 classes: aminoglycosides, β-lactams, macrolides, MDR, phenicol, and tetracycline.

One of the key objectives of this study was to determine (and quantify) the effect of the RWA production approach on the prevalence of ARGs over time in the gut microbiome of piglets as well as in manure from the barn. To that end, we compared the resistome profiles from the WGS-analyzed data, yielding frequencies of ARGs represented by the absolute number of reads corresponding to the ARGs present in the samples, which were found to belong to six main classes: aminoglycosides (18–28%), β-lactams (2–8%), macrolides (16–31%), phenicol (0.01–1.2%), multi-drug resistance (MDR; 3–7%) and tetracycline (37–45%). Overall, RWA practices reduced ARG-frequency in piglet feces (*p* = 0.017) and in-barn manure (*p* = 0.002) (Figure 4). In RWA-piglet feces, the frequency of ARGs was significantly reduced for aminoglycosides (*p* = 0.0055), macrolides (*p* = 0.02), and phenicol and tetracycline (*p* < 0.0001), relative to feces from animals raised under non-RWA conditions (Figure 5A). In RWA-barn manure, the frequency of resistance genes coding for β-lactams (*p* = 0.0046), phenicol (*p* < 0.001), MDR (*p* = 0.001), and tetracycline (*p* = 0.0034) were significantly reduced, relative to manure from non-RWA barns (Figure 5B).

**Figure 6:**
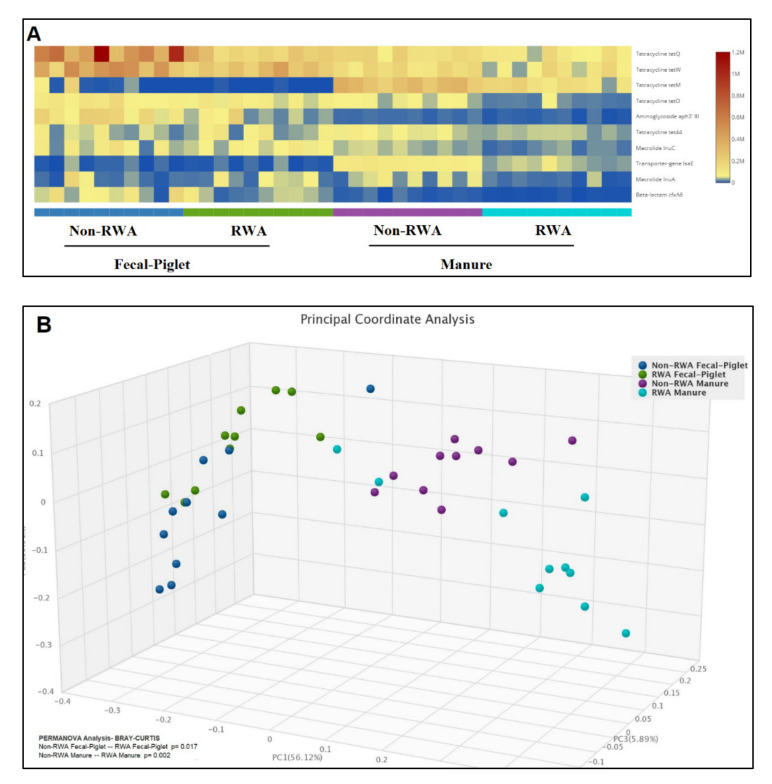
Gene frequency heat map comparative analysis of piglet feces and manure from non-RWA vs. RWA barns, showing the frequencies of the 10 most abundant ARG taxa (**A**). PCoA and ordination permANOVA analysis of the non-RWA vs. RWA resistome profiles from piglet-fecal and manure samples (**B**).

Comparative gene frequency (heat map) analysis of ARG clusters from non-RWA vs. RWA barns showed the 10 most abundant AMR taxa, wherein tetracycline resistance genes *tetQ* and *tetW* were most frequently found in all samples, followed by *tetO* in piglet feces and then *tetM* in manure (Figure 6A). Furthermore, PCA ordination of ARGs significantly clustered the effect of RWA on the resistomes from piglet feces (*p* = 0.017) and barn manure (*p* = 0.002) (Figure 6B).

## 3. Discussion

As of January 2021, the Canadian hog industry had a total inventory of about 14.02 million hogs, including 5.12 million piglets, 7.64 million grower–finishers, and 1.24 million sows and gilts [13]. However, most reports of antimicrobial usage in the Canadian swine industry are mainly focused on the grower–finisher stage, and data on piglets and sows are not well-documented [8,11]. Hence, we focused on piglets at the animal production stage to examine the effect of RWA practices on the gut microbiome and associated ARGs. From barn records that were collected over the 28-month study period, RWA and non-RWA piglets that received antibiotic treatment constituted 81% of the total number of antibiotic-treated animals, whereas grower–finishers and sows made up 17% and 2% of the total antibiotic-treated animals, respectively. This represented a total treated body mass of 95 tons for piglets, 120 tons for grower–finishers, and 113 tons for sows. 

A study conducted in the USA by Davies et al. (2020), reporting all antibiotic treatments between 2016 and 2017 from nine participating non-RWA pig barns, showed that 60% of antibiotic treatments were allocated to nursery pigs (3–10 weeks of age) and 40% to finishing pigs [14]. The authors also found that among the various antimicrobial agent classes (including tetracyclines, lincosamides, pleuromutilins, β-lactams, aminoglycosides, macrolides, and quinolones) that were used to treat pigs from weaning to market in the USA, 60% of the total drugs used were tetracyclines, 8% β-lactams, 3.5% aminoglycosides, and 3% macrolides. This contrasts with the Canadian practices shown in our study where nursery animals were treated less with tetracyclines (25% for non-RWA and 4% for RWA), while more prevalent treatments included antifolates for non-RWA (64%) and β-lactams for RWA (54%). Furthermore, drug delivery routes in the USA operations were either by feed, water, or injection, whereas Canadian drug administration was predominantly by injection [12,14]. Our drug-use data showed a 9-fold overall reduction in the amount of antibiotics used for treating piglets in RWA barns compared to non-RWA barns, where these drugs were used mainly for the treatment of limping, scours, and several other classes of injury. Although β-lactam and tetracycline resistance have been shown to be globally prevalent in pig microbiomes [15,16,17], these drug classes remain in use and clinically valuable, even in herds where ARGs are abundant across the microbiome [15,18]. Antifolates, however, which are antibacterial, immunomodulating, and chemotherapeutic agents, are widely used in Canadian swine production, but the mechanism of resistance is less well-documented and likely related to inducing efflux multidrug-resistance [19,20].

In the RWA barns included in this study, sick animals requiring antibiotics were immediately segregated from other pigs and subsequently marketed as conventional non-RWA pigs; accordingly, they were also excluded from the WGS sampling and analysis performed in this study. 

The metagenomic results from the gut microbiome of RWA pigs demonstrated a substantial reduction in ARGs, including a significantly lower frequency of resistance genes coding for aminoglycosides (*p* = 0.0055), macrolides (*p* = 0.02), and phenicol and tetracycline (*p* < 0.0001) (Figure 5A). Since these RWA pigs received no drugs throughout the surveillance period of this study (as well as for several years in advance of our study’s monitoring period), the higher frequency of ARGs observed over time in non-RWA pigs could be attributed to the administered drugs, and this, therefore, provides a direct correlation between antibiotic drug usage and AMR occurrence in the gut of piglets.

The composite manure samples analyzed in this study were collected from the in-barn manure transfer pit, where the manure from all rooms in the barn is accumulated and stored temporarily prior to being pumped to the external storage structures (e.g., lagoons) [21,22]. The differences in manure nutrient composition between swine raised under RWA versus non-RWA practices are not yet well-documented [21,23]. However, in this study, a substantial reduction in the frequency of ARGs coding for β-lactams, phenicol, MDR, and tetracycline resistance was observed in RWA manure compared to non-RWA manure samples. In addition to residual components from animal-metabolized antibiotics, several other factors could affect manure composition and potentially impact detection of AMR, including the feed program, nutrient excretion, drinking water consumption, barn water use, bedding, and climatic conditions [24]. 

Compared to non-RWA barns, the frequency of tetracycline ARGs in RWA barns was reduced (*p* < 0.05) in both piglet gut and barn manure, suggesting that RWA measures could offer an effective ‘barn-level’ mitigation strategy for AMR related to tetracycline (a drug still in use in conventional hog operations). In addition, RWA barns also showed a significant reduction in β-lactam-ARGs and MDR-ARGs, which can be associated with the higher usage of various drug classes in conventional (non-RWA) barns (e.g., β-lactams and antifolates). MDR mechanisms have often been associated with efflux-pump-based resistance in order to expel structurally unrelated drugs; this includes the antifolate and β-lactam efflux transporters [25,26].

The ‘pathome’, which represents the occurrence of pathogens in the microbiome, was extracted from our metagenomic analyses. In the available literature, no ‘user-friendly workflow pipeline’ has been developed to examine possible linkages between drug usage and pathogen prevalence in hog barns. Our analyses showed that more pathogenic *Firmicutes* bacteria were detected in RWA samples than in non-RWA samples. This suggests that the reduced usage of antibiotics inversely correlates with an increased prevalence of some pathogens. The impact of this finding on the prevalence of virulence factors and/or bacteriophages has yet to be determined. For instance, reduced antibiotic use in animals could potentially lead to the propagation of untreated pathogenic organisms in the pigs and may become a potential threat to food safety if disseminated in meat products [27,28,29], or which may lead to increased health complications within swine herds. However, it is challenging to verify possible relationships or co-occurrences of pathogens and the effect of such a negative correlation found between drug usage and the pathome. Escudeiro et al. (2019) demonstrated the presence of a positive relationship between the resistome and virulence factors present in the human gut and different environmental biome samples [6]. Accordingly, they compared microorganisms from environmental and gut samples obtained from diverse, worldwide human populations, showing that the existence of metagenome protein family richness was greater than either the resistome or virulome compositions. The authors reported that there was a correlation between pathogenicity and virulence and that it co-occurred across all types of human gastric and environmental microcosm samples. However, they performed correlations of the metagenomic richness (diversity) of the data and not the frequency of each gene read, as we describe here. The strength of our approach is its potential for revealing deeper insights into the effect of RWA practices, with quantitatively-measurable readouts and specific measurements and their correlations. In our study, we found less AMR in RWA barns, which suggests a negative correlation in RWA between the pathome and the resistome; however, this is yet to be verified.

Consistent with our findings in piglet gut, our WGS analysis demonstrated that some pathogenic *Proteobacteria* were more prevalent in the RWA manure. However, the majority of these *Proteobacteria* were likely not of pig origin since these were found in WGS data from the soil collected from the barns (data not shown) and not detected in the pig fecal samples. This could suggest that the reduction of antibiotics and their residual components in RWA barns resulted in the detection of a greater number of pathogenic *Proteobacteria* that arose from unknown environmental sources.

## 4. Materials and Methods

### 4.1. Experimental Design and Sample Collection

Our methodology included the regular collection of metadata comprised of all records of administered antibiotic drugs and illnesses or treatment reasons from two types of participating barns, RWA and non-RWA barns. Physical samples from the animals and the barn environment were also periodically collected from each barn and then sequenced and metagenomically compared.

A 3-year longitudinal research project, with 5 regular animal and barn environment sampling timepoints (at 6-month intervals) along with 28 months of drug record reporting, was conducted. Accordingly, fresh fecal samples were aseptically collected from three 6-week piglets and stored in 50 mL sterile tubes and immediately transported at 4 °C to the laboratory for storage at −80 °C and subsequent analyses. Composite manure samples were similarly collected every 6 months from the in-barn manure transfer pit. The transfer pit represents the central collection point for manure from all rooms in the barn prior to pumping out into external manure storage. Sample handling was conducted in accordance with the CDC’s Biosafety in Microbiological and Biomedical Laboratories (BMBL) manual for Level 1 materials [30].

### 4.2. Whole-Genome Sequencing and Sequence Analyses

To identify the total ARGs (resistome) and bacterial-related diversity as well as the prevalence of pathogens in the collected samples, random shotgun next-generation sequencing (NGS) was performed using an Illumina HiSeq platform (Omega-Bioservices, Norcross, GA, USA). As described previously [12], samples were handled according to sequencing service procedures and then shipped to Omega-Bioservices for DNA extraction, data quality determination, and NGS. Briefly, DNA was extracted from 1 g of sample material using the Mag-Bind Universal Pathogen DNA Kit (Omega Bio-tek, Inc. Norcross, GA, USA), and the purity and yield of the DNA were verified using the Quant-iT™ PicoGreen™ ds DNA System Kit (ThermoFisher Scientific, Pittsburgh, PA, USA). Shotgun NGS libraries were constructed from DNA using the Kapa Biosystems Prep Kit, following the manufacturers’ protocols (Roche^®^, KK2103 Pleasanton, CA, USA). Samples representing a distinct time point were run on one lane of a HiSeq4000/X Ten (Illumina) instrument, generating a total of 100–120 GB of 150-bp paired-end data reads. Eight samples per run produced an average minimum of ~30 million reads (MReads) per sample, with each sample generating 2 FASTQ files (R1 Forward read and R2 Reverse read) shared through the BaseSpace Sequence Hub. Sequences were then subjected to quality control processes (i.e., denoising and trimming the adaptors) and reported with the MultiQC tool (https://multiqc.info/ accessed on 23 March 2021) prior to uploading onto the platform for metagenomic analysis (CosmosID Inc., Rockville, MD, USA). As previously described, CosmosID focuses on the rapid gene-marker characterization of multiple genomic features, including microorganisms and antimicrobial resistance, for infectious disease identification, food safety inspections, pharmaceutical discovery, public health surveillance, and microbiome analysis [12,31,32].

Recently, we detailed a workflow method of a health metadata-based management approach for comparing and quantifying WGS data targeting the prevalence of pathogens and antimicrobial resistance reduction in Canadian hog barns [12]. Based on that methodology, described in brief here, 8 samples per run generated between 20 and 100 million reads (MReads) per sample, for a total number of reads ranging from 1000 to 1200 MReads. For comparative analyses among the non-RWA and RWA samples, all data sets were subsampled to a fixed 20 MRead depth to ensure uniform population diversity and reduce bias in the data analyses arising from variations in read depth. The subsampling method involved rarefying randomly sampled reads without replacement from each of the 8 samples, up to the common count of 20 MReads, using the seqtk tool package, available at https://github.com/lh3/seqtk accessed on 23 March 2021. The rarefaction depth from each run was set to the lowest MReads per sample that provided total coverage (20 MReads), with the remaining reads being discarded [33]. Consequently, the difference in the number of reads obtained from different samples reflected the biological differences present in the samples. The metagenomic shotgun analysis employs a functionality-based strategy, wherein functional gene products are identified regardless of which bacterial/microbial species the genetic material originates from. This read-based profiling method allows the multiple profiling of targets, including those based on taxonomic, resistome, and virulome criteria. In this procedure, all our unassembled sequencing reads were analyzed using the CosmosID software package, which utilizes data mining algorithms and curated databases that provide fine resolution for organism identification and discrimination at the strain level as well as genes of interest, along with accurate measurement of their frequency and relative abundances.

### 4.3. Profiling the Prevalence of Pathogens

The prevalence of pathogens (the pathome) was identified using a subset of the taxonomic profiles corresponding to bacterial pathogens. First, we determined taxonomic profiles through microbial identification to the species, subspecies, and strain levels, along with the quantification of the identified organism’s relative abundance and frequency at each taxonomic level through GenBook comparators and the GENIUS software implemented within the CosmosID algorithm. The pathome subset then included only pathogenic bacteria identified at the strain level. The table listing of bacterial species, obtained from taxonomy profiling, was further used for manual determination of human and animal risk groups (RG1, RG2, or RG3). The bacterial species lists were first queried against animal and human RG databases, including (1) the RG and Risk Assessment database hosted by the Public Health Agency of Canada (https://health.canada.ca/en/epathogen accessed on 3 May 2021), (2) the Bacterial Diversity Meta-database (https://bacdive.dsmz.de accessed on 3 May 2021), (3) the RG database hosted by the Association for Biosafety and Biosecurity (https://my.absa.org/Riskgroups accessed on 3 May 2021), and (4) the GESTIS Biological Agents Database (https://bioagent.dguv.de/search accessed on 3 May 2021). The pathome profiles represented all identified organisms classified as risk groups, other than RG Level 1.

### 4.4. Profiling the Resistome

The collection of ARGs in the microbiome (the resistome) was also profiled by querying unassembled sequence reads against CosmosID’s curated ARG database, generating a tabular list of identified and quantified ARGs. The ARG database in the platform utilizes multiple inputs, including NCBI- RefSeq, PATRIC, M5NR, ENA, DDBJ, CARD, ResFinder, ARDB, and ARG-ANNOT. Together, these databases comprise over 4000 identifiable ARGs based on the percent gene coverage for each gene as a function of the gene-specific read frequency in each sample. The resultant ARG profile table was then clustered into 16 classes of drug resistance and 7 mechanisms of resistance [12] based on a classification system combined from two pipelines, with a focus on antimicrobial resistance (https://megares.meglab.org/ and https://card.mcmaster.ca/ accessed on 3 May 2021). 

### 4.5. Statistical Analysis

Diversity, ordination, and differential frequency, as well as both multivariate and univariate analyses, were applied to the resultant taxa/ARG frequency tables. These were used to calculate observed and expected species richness (Shannon alpha diversity indices and beta diversity distance matrices). For statistical analysis and equally distributed comparisons, we included data from two of each type of farm (2 non-RWA and 2 RWA), which were considered two biological replicates for each type. Samples were collected repeatedly over 5 time points at 6 month intervals. For the two readout features, i.e., pathogen species and ARG class, principal coordinate analysis (PCoA) was performed to cluster samples based on their occurrence frequency (Bray-Curtis distance matrix; community structure). Significantly different features were identified using permANOVA analysis. Individual features, such as individual ARG classes of drug resistance, were compared using 2-way parametric ANOVA, with ‘non-RWA’ and ‘RWA’ as barn groups and ‘fecal’ and ‘manure’ and ‘time-point repeated measurements’ as subgroups.

### 4.6. Data Availability

DNA metagenomic sequencing data are available in the Sequence Read Archive (SRA) (https://submit.ncbi.nlm.nih.gov/subs/sra/ accessed on 3 May 2021) under accession number PRJNA737271.

## 5. Conclusions

Due to new, more stringent regulations on the use of antibiotics in livestock production, producers have adopted practices such as the raised without antibiotics (RWA) approach to try to reduce or eliminate the use of antibiotics and to mitigate the emergence, selection, and spread of antimicrobial resistance (AMR). In this study, we used next-generation sequencing as an alternative to phenotypic susceptibility testing. The surveillance of prevalent antimicrobial resistance genes (ARGs) to quantitatively assess the effect of RWA on AMR showed a potential positive correlation between RWA practices and reduced frequency of ARGs in piglet gut and manure samples. However, the absence of antibiotic usage in RWA farms appears to have resulted in the detection of more pathogens. Overall, we have demonstrated that raising pigs without antibiotics impacts the prevalence of pathogens and a specific set of AMR classes over time.

These findings can contribute to new efforts to develop intervention measures or revise existing RWA practices targeting pigs in early development stages, aimed at reducing both AMR and persistent pathogens. This aligns with the overall priority of reducing total antibiotic usage while curbing the development and spread of AMR related to animal agriculture.

## Data Availability

DNA metagenomic sequencing data are available in the Sequence Read Archive (SRA) (https://submit.ncbi.nlm.nih.gov/subs/sra/ accessed on 23 March 2021) under accession number PRJNA737271.

## Supplementary Materials

The following are available online at https://www.mdpi.com/article/10.3390/antibiotics10101152/s1. Figure S1: Stacked bars taxonomy profiling at the phylum level; Table S1: Summary of the number of piglets, type and quantity of antibiotics, and reasons for piglet. Table S2. Pathome–List of pathogens detected in piglet feces. Table S3. Pathome–List of pathogens detected in manure.
