# Supplementary material for: Piglet Gut and in-Barn Manure from Farms on a Raised without Antibiotics Program Display Reduced Antimicrobial Resistance but an Increased Prevalence of Pathogens
Source: Antibiotics (Basel). 2021 Sep 24;10(10):1152. doi: 10.3390/antibiotics10101152 (PMC8532630; doi:10.3390/antibiotics10101152)
Supplement: Supplementary file 1 [file antibiotics-10-01152-s001.zip › antibiotics-1332335-supply.pdf]

Table S1. Summary of the number of piglets, type and quantity of antibiotics, and reasons for piglet antibiotic treatments in the participating barns.

Numbers of piglets	Type of antibiotics	Reason for treatment						Quantity of antibiotics used	
		Infection	Injury	Limping	Poor-doers	Respiratory	Scours	mg	percent/type
**Non-RWA**									
Piglets (N=14,312)	Antifolates	19,176	84,408	1,511,155	144,000	102,450	1,516,238	3,377,428	64%
	β-lactams	343,770	1,058	95,645	6,920	18,993	119,860	586,245	11%
	Tetracycline	0	600	2,000	5,700	300	1,322,540	1,331,140	25%
Total antibiotics (mg)		362,946	86,066	1,608,800	156,620	121,743	2,958,638	**5,294,813**	
Antibiotics percent/reason		7%	2%	30%	3%	2%	56%		
**RWA**									
Piglets (N=1,697)	Antifolates	3,936	22,020	193,524	33,108	516	2,880	255,984	30%
	β-lactams	81,770	245,685	130,820	1,275	900	1,800	462,250	54%
	Chloramphenicol	6,075	5,175	98,700	900	0	0	110,850	13%
	Tetracycline	1,600	16,350	11,950	1,100	750	0	31,750	4%
Total antibiotics (mg)		93,381	289,230	434,994	36,383	2,166	4,680	**860,834**	
Antibiotics percent/reason		11%	34%	51%	4%	0%	1%		

Table S2. Pathome – List of pathogens detected in piglet feces.

					nonRWA Fecal Piglet					RWA Fecal Piglet				
**Phylum**	**Class**	**Specie-Strain**	**RG Human**	**RG Animal**	**43405**	**43586**	**43770**	**43952**	**44136**	**43405**	**43586**	**43770**	**43952**	**44136**
**Proteobacteria**	**γproteobacteria**	Proteus hauseri ZMd44	RG2	RG1	0	2263	0	0	0	0	2263	0	0	0
		Proteus vulgaris	RG2	RG1	0	2317	0	0	0	0	2317	0	0	0
		Proteus penneri ATCC 35198	RG2	RG1	0	1774	0	0	0	0	1774	0	0	0
		Providencia rettgeri	RG2	RG1	0	13	0	0	0	0	13	0	0	0
		Morganella morganii H1r	RG2	RG2	0	1317	0	0	0	0	1317	0	0	0
		Proteus mirabilis ATCC 29906	RG2	RG2	0	353	0	0	0	0	353	0	0	0
		Salmonella enterica subsp. enterica serovar Enteritidis	RG2	RG2	0	0	140	0	0	0	0	0	0	0
		Shigella sonnei	**RG2**	**RG2**	0	0	0	0	0	0	0	0	0	30
		Shigella dysenteriae	RG2	RG2	0	0	0	0	0	0	0	0	0	33
		Shigella flexneri MT1457	RG2	RG2	0	252	0	310	0	0	252	0	0	57
	**βproteobacteria**	Sutterella wadsworthensis	RG2	RG1	24	0	10	0	0	35	0	56	36	26
	**δproteobacteria**	Bilophila wadsworthia 3_1_6	RG2	RG2	0	934	0	0	0	0	934	0	0	0
	**εproteobacteria**	Helicobacter pullorum MIT 98-5489	RG2	RG2	798	0	0	0	0	0	0	0	0	0
		Campylobacter coli BIGS0005	RG2	RG2	0	0	677	0	145	0	0	59	287	56
		Helicobacter canadensis MIT 98-5491	RG2	RG1	0	0	0	0	0	0	0	0	0	789
		Helicobacter rodentium ATCC 700285	RG1	RG2	6643	0	0	0	0	0	0	0	0	0
**Bacteroidetes**	**Bacteroidia**	Parabacteroides distasonis str. 3999B T(B) 4	RG2	RG1	0	18	0	0	903	0	18	1748	9064	7415
		Alistipes senegalensis JC50	RG2	RG2	0	0	0	11642	0	0	0	0	0	0
		Prevotella albensis DSM 11370 = JCM 12258	RG2	RG1	17166	4430	9784	10024	12670	9979	4430	5034	7698	7125
		Prevotella bivia	RG2	RG2	2811	1957	4336	3674	393	3284	1957	764	2568	1712
		Prevotella melaninogenica DNF00666	RG2	RG2	6566	3531	3025	6548	18429	8927	3531	4939	6002	3875
		Prevotella oris	RG2	RG1	999	0	0	1166	0	1122	0	0	0	0
		Prevotella buccalis DNF00853	RG2	RG2	4907	3468	4367	4374	0	0	3468	0	0	0
		Prevotella buccae ATCC 33574	RG2	RG2	0	0	0	0	0	1534	0	0	0	0
		Bacteroides caccae ATCC 43185	RG2	RG2	679	0	0	0	0	0	0	0	0	0
		Bacteroides pyogenes DSM 20611 = JCM 6294	RG1	RG2	1248	301	927	2035	1635	2258	301	1187	855	1159
		Prevotella brevis ATCC 19188	RG2	RG1	9672	4060	4840	5201	6383	12511	4060	6406	9889	12543
		Prevotella baroniae	RG2	RG2	2111	0	3638	2418	3275	2323	0	0	0	1655
		Prevotella denticola	RG2	RG2	1227	0	1052	0	2044	1549	0	0	0	882
		Prevotella disiens DNF00882	RG2	RG1	1175	0	0	0	0	1121	0	0	0	0
		Prevotella multisaccharivorax DSM 17128	RG2	RG2	3601	0	0	0	6224	3669	0	0	0	3074
		Bacteroides fragilis HMW 615	RG2	RG2	6574	3722	4836	8077	8096	8762	3722	4531	3251	5319
		Prevotella bryantii	RG2	RG1	4055	1763	1690	3631	4746	4135	1763	1997	3129	2612
		Prevotella salivae F0493	RG2	RG1	0	0	0	6129	0	0	0	9492	13160	0
		Prevotella bergensis DSM 17361	RG2	RG2	0	0	0	0	5958	0	0	0	0	0
		Bacteroides ovatus CL02T12C04	RG2	RG2	0	0	0	0	6523	0	0	0	0	3519
		Bacteroides faecis MAJ27	RG2	RG2	0	0	0	0	70795	0	0	0	0	0
		Bacteroides thetaiotaomicron VPI-5482	RG2	RG1	0	4470	0	0	0	0	4470	0	0	0
		Bacteroides eggerthii	RG2	RG2	0	0	0	1542	1403	0	0	0	0	0
		Bacteroides vulgatus PC510	RG2	RG1	2267	0	0	0	0	0	0	0	0	0
		Bacteroides uniformis ATCC 8492	RG2	RG1	7168	14978	0	0	3784	0	14978	0	0	0
	**Negativicutes**	Megasphaera elsdenii	RG2	RG1	34141	48830	362696	79476	0	47507	48830	233449	248652	0
		Acidaminococcus fermentans DSM 20731	RG2	RG1	79140	21136	535111	111064	1956	16840	21136	77228	462917	100674
		Acidaminococcus intestini RyC-MR95	RG2	RG2	0	0	0	7216	0	0	0	0	19979	5948
		Mitsuokella multacida DSM 20544	RG2	RG2	21162	89246	215224	264707	9202	3369	89246	7060	1169323	25299
		Aerococcus viridans LL1	RG2	RG2	819	37929	0	0	0	0	37929	10	0	0
		Enterococcus avium ATCC 14025	RG2	RG1	0	4035	0	0	0	0	4035	0	0	0
		Enterococcus faecium	RG2	RG1	0	3574	0	0	0	484	3574	169	0	0
		Enterococcus faecalis EnGen0413	RG2	RG2	0	116	0	0	0	0	116	0	0	0
		Enterococcus hirae ATCC 9790	RG2	RG2	0	465	0	0	0	0	465	0	0	0
		Enterococcus villorum ATCC 700913	RG1	RG2	0	11968	0	0	0	0	11968	0	0	0
		Lactobacillus crispatus EM-LC1	RG2	RG2	0	0	0	0	0	0	0	541	0	0

**Firmicutes**	**Bacilli**	Streptococcus caballi DSM 19004	RG2	RG2	0	3297	0	0	0	0	3297	0	0	0
		Streptococcus gallolyticus subsp. gallolyticus	RG2	RG2	0	7478	2495	3264	1031	163	7478	2001	8631	92
		Streptococcus equinus JB1	RG2	RG2	0	8524	1291	3336	1185	1391	8524	5277	13617	0
		Streptococcus equi subsp. zooepidemicus BHS5	RG2	RG2	0	2137	3279	830	677	689	2137	0	733	647
		Streptococcus agalactiae	RG2	RG2	196	138	385	73	126	101	138	22	82	0
		Streptococcus infantarius subsp. infantarius	RG1	RG2	171	2434	841	1755	380	273	2434	1190	5447	483
		Streptococcus lutetiensis 033	RG2	RG2	991	33886	12325	16173	5716	2663	33886	11261	50561	3174
		Streptococcus parauberis	RG1	RG2	0	399	0	0	0	0	399	0	0	0
		Streptococcus pneumoniae 1488	RG2	RG2	0	0	0	0	0	0	0	0	0	0
		Streptococcus pasteurianus ATCC 43144	RG2	RG2	0	1165	499	541	0	0	1165	0	0	0
		Streptococcus suis GZ1	RG2	RG2	5213	5101	11869	9887	5740	3595	5101	5420	1966	1986
	**Tissierellia**	Anaerococcus prevotii	RG2	RG1	0	13	0	0	0	0	13	0	0	0
		Finegoldia magna	RG2	RG2	0	82	0	0	0	0	82	0	0	0
	**Clostridia**	Erysipelothrix rhusiopathiae	RG2	RG2	0	92	0	0	0	0	92	0	0	0
		Pseudoflavonifractor capillosus ATCC 29799	RG2	RG2	29283	0	0	7213	36960	14422	0	3250	0	7676
		Faecalibacterium cf. prausnitzii KLE1255	RG2	RG2	0	0	0	0	108835	0	0	135186	0	157946
		Flavonifractor plautii ATCC 29863	RG2	RG2	4124	2929	1446	2057	2456	1811	2929	787	96	38
		Clostridium botulinum	RG2	RG2	0	0	0	0	0	0	0	0	0	0
		Clostridium butyricum	RG2	RG2	61	48652	128	19443	34521	34147	48652	13549	26907	14672
		Clostridium cadaveris AGR2141	RG2	RG1	0	1443	0	0	0	0	1443	0	0	0
		Clostridioides difficile 6534	RG2	RG2	1287	1026	4998	772	0	1115	1026	833	2022	0
		Paeniclostridium sordellii	RG2	RG2	0	191	0	388	0	0	191	371	0	0
		Peptostreptococcus anaerobius	RG2	RG2	0	1116	0	0	0	0	1116	0	0	0
		Terrisporobacter glycolicus ATCC 14880 = DSM 1288	RG2	RG2	0	3992	0	3040	1607	0	3992	4502	0	0
		Clostridium orbiscindens 1_3_50AFAA	RG2	RG2	0	0	1006	0	914	0	0	0	0	798
		Clostridium perfringens C str. JGS1495	RG2	RG2	0	2094	98	496	204	0	2094	274	0	0
		Eubacterium ventriosum ATCC 27560	RG2	RG2	0	0	0	0	0	0	0	0	0	3163
		Clostridium symbiosum	RG2	RG1	0	0	0	0	0	0	0	0	0	13
		Catonella morbi ATCC 51271	RG2	RG1	3523	0	0	0	0	0	0	0	0	0
**Actinobacteria**	**Actinobacteria**	Trueperella pyogenes TP8	RG2	RG2	0	1231	0	0	0	0	1231	0	0	0
		Bifidobacterium sp. AGR2158	RG2	RG1	0	0	11062	0	0	0	0	0	0	0
	**Coriobacteriia**	Olsenella uli	RG2	RG2	0	723	2160	988	0	0	723	0	0	0
		Collinsella aerofaciens ATCC 25986	RG2	RG1	0	0	0	31832	10960	0	0	16948	48193	16933
		Eggerthella lenta	RG2	RG2	0	399	0	0	0	0	399	0	0	0
**Chlamydiae**	**Chlamydiia**	Chlamydia suis MD56	RG2	RG2	0	80686	716	45601	64283	1293	80686	60192	1112	14308
		Chlamydia muridarum MopnTet14	RG1	RG2	0	355	0	0	0	0	355	655	0	0
**Spirochaetes**	**Spirochaetia**	Brachyspira murdochii DSM 12563	RG2	RG2	0	0	0	0	0	0	0	0	4849	0
**Fusobacteria**	**Fusobacteriia**	Fusobacterium varium ATCC 27725	RG2	RG2	0	791	0	0	0	0	791	0	0	0
		Fusobacterium necrophorum	RG2	RG2	0	6	0	0	0	0	6	0	0	0
	**Total**				259793	479583	1206943	676912	440152	191065	479583	616381	2050840	405722

Table S3. Pathome – List of pathogens detected in manure.

Phylum	Class	Specie-Strain	RG Human	RG Animal	nonRWA Manure					RWA Manure				
					Nov-18	May-19	Nov-19	May-20	Nov-20	Nov-18	May-19	Nov-19	May-20	Nov-20
Proteobacteria	α-proteobacteria	Paracoccus sanguinis	RG2	RG2	1	0	0	0	0	411	0	0	0	65
	γ-proteobacteria	Escherichia coli O157:H7 str. 2011EL-2	RG3	RG3	1	0	0	0	0	1	0	0	0	1221
		Acinetobacter baumannii 573719	RG2	RG1	1	224	153	153	102	2182	224	0	26	32
		Acinetobacter johnsonii	RG1	RG2	1	0	261	0	0	1	0	0	0	333
		Acinetobacter junii	RG2	RG2	1	0	0	0	0	1	0	0	0	24
		Actinobacillus minor	RG2	RG2	1	0	0	0	0	1	0	216	0	0
		Acinetobacter lwoffii NCTC 5866 = CIP	RG2	RG1	1	0	0	0	830	1	0	0	0	1858
		Escherichia fergusonii	RG2	RG2	1	0	684	0	0	1	0	0	0	0
		Proteus vulgaris	RG2	RG1	1	0	0	0	0	2214	0	0	0	0
		Proteus penneri ATCC 35198	RG2	RG1	1	0	0	0	0	1977	0	0	0	0
		Providencia alcalifaciens	RG2	RG1	1	0	0	0	0	1	0	0	0	15
		Providencia rettgeri	RG2	RG1	1	613	0	0	0	1419	613	30	0	0
		Pseudomonas aeruginosa	RG2	RG2	1	45	0	0	73	5746	45	0	97	4841
		Psychrobacter phenylpyruvicus DSM 7	RG2	RG1	1	1942	1580	0	2080	6537	1942	2005	0	0
		Leclercia adecarboxylata	RG2	RG1	1	0	0	0	0	1	0	0	0	109
		Salmonella enterica subsp. enterica se	RG2	RG2	1	0	0	0	0	1	0	0	0	34
		Proteus mirabilis	RG2	RG2	1	0	0	0	0	3199	0	0	0	0
		Serratia liquefaciens	RG1	RG2	1	0	0	0	0	1	0	0	590	2692
		Serratia marcescens	RG2	RG2	1	0	0	0	0	1	0	0	184	1979
		Vibrio cholerae CP1035(8)	RG2	RG1	1	0	0	0	0	323	0	0	192	216
		Yersinia enterocolitica	RG2	RG2	1	0	22	0	0	1	0	0	0	0
	β-proteobacteria	Sutterella wadsworthensis	RG2	RG1	8	0	0	8	0	5	0	0	10	1489
		Oligella urethralis	RG2	RG1	1	0	0	0	0	477	0	0	0	0
		Comamonas aquatica	RG2	RG2	1	1249	0	0	0	1	1249	0	0	838
		Alcaligenes faecalis subsp. faecalis NBI	RG2	RG2	1	16326	0	0	1280	11426	16326	5934	0	2330
	δproteobacteria	Bilophila wadsworthia 3_1_6	RG2	RG2	1	0	0	0	0	1	0	1022	13142	0
	εproteobacteria	Arcobacter butzleri	RG2	RG2	128	23	307	198	406	56	23	56	545	1747
		Helicobacter bilis	RG1	RG2	1	0	0	0	0	1	0	0	0	180
		Campylobacter coli BIGS0005	RG2	RG2	78	0	114	0	42	1	0	0	0	39
		Helicobacter rodentium ATCC 700285	RG1	RG2	3325	0	1293	713	0	1	0	0	0	0
Bacteroidetes	Bacteroidia	Parabacteroides distasonis str. 3999B	RG2	RG1	1357	1229	2393	0	1600	0	1229	200	276	459
		Porphyromonas asaccharolytica DSM 1	RG2	RG2	653	0	595	613	1263	0	0	670	0	0
		Porphyromonas gulae	RG1	RG2	0	0	0	4566	9333	0	0	1133	0	1669
		Prevotella albensis DSM 11370 = JCM	RG2	RG1	0	2109	0	0	0	0	2109	0	0	0
		Prevotella bivia	RG2	RG2	458	918	723	0	694	0	918	0	0	0
		Prevotella melaninogenica DNF00666	RG2	RG2	1872	1860	2659	0	5243	0	1860	2187	0	0
		Bacteroides caccae ATCC 43185	RG2	RG2	0	0	0	0	0	0	0	0	1128	885
		Bacteroides pyogenes DSM 20611 = J(	RG1	RG2	106	2156	333	127	268	107	2156	1594	316	152
		Prevotella brevis ATCC 19188	RG2	RG1	810	1957	1126	0	556	0	1957	470	298	1109
		Prevotella bergensis DSM 17361	RG2	RG2	0	0	0	0	0	0	0	4498	0	0
		Prevotella multisaccharivorax DSM 17	RG2	RG2	0	0	0	0	5568	0	0	0	0	0
		Odoribacter splanchnicus DSM 20712	RG2	RG1	0	0	0	0	0	0	0	5321	6342	0
		Sanguibacteroides justesenii	RG2	RG2	0	0	0	0	0	0	0	1717	533	0
		Bacteroides fragilis HMW 615	RG2	RG2	1672	1431	1639	865	1011	499	1431	665	8225	737
		Prevotella bryantii	RG2	RG1	623	568	870	220	776	0	568	314	0	173
		Prevotella salivae F0493	RG2	RG1	0	2328	0	0	0	0	2328	0	0	0
		Bacteroides nordii WAL 11050 = JCM 1	RG2	RG2	702	1350	871	0	0	0	1350	0	0	0

		Bacteroides ovatus SD CMC 3f	RG2	RG2	0	0	0	0	4758	0	0	1361	645	2398
		Bacteroides uniformis ATCC 8492	RG2	RG1	1006	843	418	279	901	115	843	163	136	254
		Bacteroides eggerthii	RG2	RG1	0	0	0	0	0	0	0	0	0	1552
		Myroides odoratimimus CCUG 12901	RG2	RG1	0	0	0	0	0	1077	0	251	29	0
	**Flavobacteriia**	Myroides odoratus	RG2	RG1	0	0	0	0	0	0	0	0	0	1079
		Empedobacter falsenii	RG2	RG1	0	0	0	0	1287	0	0	0	0	5030
		Empedobacter brevis NBRC 14943 = A	RG2	RG2	0	1991	0	0	0	0	1991	0	0	0
		Megasphaera elsdenii	RG2	RG1	19522	23007	36687	24104	0	3431	23007	15957	5449	0
		Bacteroides nordii	RG2	RG1	3218	0	0	0	0	0	0	0	0	0
	**Negativicutes**	Acidaminococcus fermentans DSM 20	RG2	RG1	119072	20696	60000	55789	35881	4666	20696	33222	76082	5756
		Mitsuokella jalaludinii DSM 13811	RG2	RG2	36676	0	0	0	0	0	0	0	0	0
		Mitsuokella multacida DSM 20544	RG2	RG2	6268	9081	9381	4513	10267	6027	9081	6791	10395	5255
		Acidaminococcus intestini RyC-MR95	RG2	RG1	0	0	0	0	0	0	0	0	0	6617
		Aerococcus viridans LL1	RG2	RG2	11347	18548	20569	35642	11567	4806	18548	1633	3485	533
		Bacillus cereus	RG2	RG2	0	0	0	0	0	4	0	0	0	0
		Enterococcus faecium	RG2	RG1	1295	570	218	188	311	86	570	183	2464	15
		Enterococcus faecalis EnGen0413	RG2	RG2	1859	171	707	503	663	546	171	278	119	204
		Enterococcus hirae ATCC 9790	RG2	RG2	0	573	0	120	176	0	573	0	412	0
		Jeotgalicoccus saudimassiliensis	RG2	RG2	0	0	0	0	0	0	0	0	0	0
		Lactobacillus crispatus EM-LC1	RG2	RG2	68	0	0	0	0	0	0	0	0	0
		Lactococcus garvieae	RG1	RG2	0	176	0	0	209	353	176	198	41	0
		Listeria monocytogenes FSL F2-515	RG2	RG2	0	0	335	0	191	0	0	0	0	0
		Staphylococcus aureus	RG2	RG2	0	0	0	0	27	0	0	14	0	0
	**Bacilli**	Staphylococcus simulans	RG2	RG2	0	2086	0	0	0	0	2086	0	0	0
		Streptococcus gallolyticus subsp. gallo	RG2	RG2	107	4596	3235	104	237	1371	4596	3609	1269	58
		Streptococcus equinus JB1	RG2	RG2	25	2652	5717	3855	3533	3250	2652	8131	3388	7
		Streptococcus equi subsp. zooepidemi	RG2	RG2	0	1208	0	0	0	0	1208	39	0	0
		Streptococcus agalactiae	RG2	RG2	0	29	25	10	37	0	29	22	0	0
		Streptococcus infantarius subsp. infan	RG1	RG2	922	2562	2937	2225	762	919	2562	2362	19596	721
		Streptococcus lutetiensis 033	RG2	RG2	6811	25889	24944	18089	13213	10844	25889	7639	7682	3517
**Firmicutes**		Streptococcus parauberis	RG1	RG2	516	790	213	173	227	0	790	0	0	0
		Streptococcus salivarius SK126	RG2	RG1	0	0	0	0	544	0	0	0	0	0
		Streptococcus pasteurianus ATCC 431	RG2	RG2	0	648	695	672	606	335	648	509	350	0
		Streptococcus suis GZ1	RG2	RG2	2026	1558	1711	1107	1360	734	1558	3867	744	3344
	**Tissierellia**	Anaerococcus prevotii	RG1	RG2	9	49	1576	1009	52	0	49	1514	94	0
		Finegoldia magna	RG2	RG2	43	339	312	94	197	246	339	47	0	0
	**Erysipelotrichia**	Erysipelothrix rhusiopathiae	RG2	RG2	0	236	0	0	97	0	236	1071	0	0
		Erysipelothrix tonsillarum DSM 14972	RG2	RG1	8249	9335	9528	8555	5809	3459	9335	10383	3267	0
		Faecalibacterium cf. prausnitzii KLE12!	RG2	RG2	0	0	0	0	23087	0	0	0	0	11351
		Flavonifractor plautii ATCC 29863	RG2	RG2	0	0	25	0	32	0	0	30	21	0
		Intestinibacter bartlettii DSM 16795	RG2	RG1	1400	0	0	0	3909	0	0	0	0	0
		Clostridium baratii str. Sullivan	RG2	RG2	0	17248	0	0	0	1555	17248	3879	3260	1961
		Clostridium botulinum	RG2	RG2	0	600	0	0	25	1778	600	119	1267	47
		Clostridium butyricum	RG2	RG2	210	2687	3295	1559	1107	1395	2687	1478	7254	105
		Clostridioides difficile 6534	RG2	RG2	532	101	0	17	19	35	101	12	2245	0
	**Clostridia**	Paeniclostridium sordellii	RG2	RG2	0	2163	687	1951	0	0	2163	501	1507	0
		Paraclostridium bifermentans	RG2	RG2	0	92	0	0	0	0	92	0	0	0
		Terrisporobacter glycolicus ATCC 1488	RG2	RG2	0	23864	5179	5563	6624	6399	23864	3902	13999	0
		Terrisporobacter othiniensis	RG2	RG2	0	0	5151	12020	0	0	0	0	333	0

		Clostridium paraputrificum AGR2156	RG2	RG1	0	3195	0	0	0	0	3195	0	0	0
		Clostridium perfringens C str. JGS1495	RG2	RG2	95	9106	1075	1321	2587	1135	9106	1417	3609	870
		Clostridium novyi A str. 4552	RG2	RG2	1847	5401	3591	3369	0	0	5401	2214	3431	0
		Clostridium tetani	RG2	RG2	261	430	596	468	31	448	430	34	26	336
		[Clostridium] clostridioforme	RG2	RG1	0	0	0	0	664	0	0	0	0	88
**Actinobacteria**	**Actinobacteria**	Trueperella pyogenes TP8	RG2	RG2	0	0	0	0	434	0	0	0	0	0
		Corynebacterium pilosum DSM 20521	RG2	RG2	0	6515	0	1285	0	5163	6515	0	0	0
		Corynebacterium freneyi DNF00450	RG2	RG2	0	34751	1773	2609	28124	29262	34751	14837	43128	31525
		Corynebacterium urealyticum	RG2	RG2	0	2318	0	0	1283	2690	2318	1075	0	351
		Dietzia cinnamea P4	RG2	RG1	0	1431	0	0	0	0	1431	0	0	0
		Mycobacterium avium subsp. avium E	RG2	RG2	0	0	0	0	0	1965	0	0	0	0
		Corynebacterium jeikeium	RG2	RG1	0	0	0	0	0	0	0	0	0	56
	**Coriobacteriia**	Atopobium vaginae	RG2	RG1	0	21	0	0	0	0	21	0	0	0
		Collinsella aerofaciens ATCC 25986	RG2	RG1	0	8952	0	0	11750	0	8952	3808	0	5412
		Eggerthella lenta	RG2	RG2	0	472	0	155	209	0	472	0	0	0
**Cyanobacteria**	**Oscillatoriophycideae**	Planktothrix agardhii NIVA-CYA 126/8	RG2	RG1	0	646	0	0	0	0	646	0	0	0
**Chlamydiae**	**Chlamydiia**	Chlamydia suis MD56	RG2	RG2	3052	2195	3768	1097	1806	670	2195	5516	660	4119
**Synergistetes**	**Synergistia**	Pyramidobacter piscolens W5455	RG2	RG1	0	0	0	0	0	0	0	36623	9003	1136
**Tenericutes**	**Mollicutes**	Acholeplasma laidlawii PG-8A	RG1	RG2	4431	0	3425	3484	2054	2184	0	1843	4042	2169
		Acholeplasma hippikon ATCC 29725	RG1	RG2	3046	0	0	0	0	0	0	0	0	0
		Acholeplasma equifetale ATCC 29724	RG1	RG2	4723	0	2180	0	0	2391	0	0	0	0
		Acholeplasma modicum ATCC 29102	RG1	RG2	0	0	0	0	13236	0	0	0	0	3508
		Acholeplasma axanthum ATCC 25176	RG1	RG2	21874	1684	8077	9605	7369	10167	1684	8158	13333	2388
**Spirochaetes**	**Spirochaetia**	Brachyspira murdochii DSM 12563	RG2	RG1	0	0	0	0	0	0	0	0	0	679
**Fusobacteria**	**Fusobacteriia**	Fusobacterium necrophorum	RG2	RG2	0	0	0	0	0	0	0	0	0	0
		Fusobacterium ulcerans	RG2	RG2	0	752	586	0	486	0	752	1164	0	1019
	**Total**				272292	288571	234228	208990	228857	145052	288571	213871	274659	128671
