# Supplementary figures and images for: Piglet Gut and in-Barn Manure from Farms on a Raised without Antibiotics Program Display Reduced Antimicrobial Resistance but an Increased Prevalence of Pathogens
Source: Antibiotics (Basel). 2021 Sep 24;10(10):1152. doi: 10.3390/antibiotics10101152 (PMC8532630; doi:10.3390/antibiotics10101152)

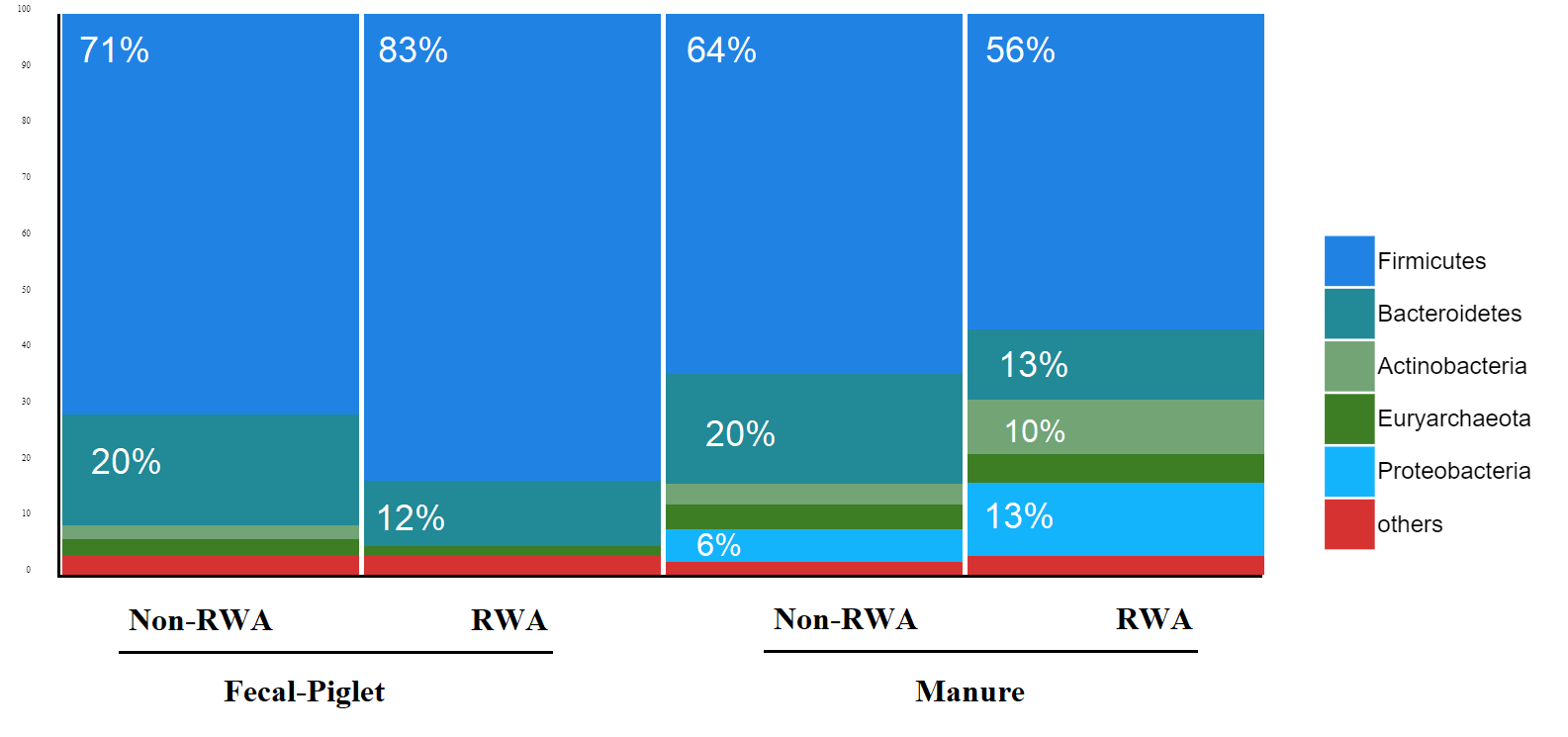

Supplement: Supplementary file 1 [file antibiotics-10-01152-s001.zip › Figure S1.png]
